# Complete Genome Sequence of a Novel Porcine Circovirus Type 3 Strain, CH/GX/1776D/2017, Isolated from Guangxi, China

**DOI:** 10.1128/genomeA.00285-18

**Published:** 2018-04-26

**Authors:** Bingxia Lu, Yibin Qin, Ying He, Lei Liu, Qunpeng Duan, Yingning Zhou, Bin Li, Zhongwei Chen, Jiaxing Liang, Qianlian Su, Bingfen Bi, Dongfu Jiang, Jingzhuan Lu, Wu Zhao

**Affiliations:** a Guangxi Key Laboratory of Veterinary Biotechnology/Department of Virology, Guangxi Veterinary Research Institute, Nanning, Guangxi, China

## Abstract

Porcine circovirus type 3 (PCV3) was first described in 2016 in U.S. swine herds as a pathogenic agent for pigs. To date, PCV3 has been reported to be widely circulating in the United States, China, South Korea, Brazil, Italy, and Poland. Here, we report the genome sequence of a PCV3 strain (CH/GX/1776D/2017) isolated from Guangxi Province in southern China. The sequence data presented in this study will help us better understand the molecular characteristics and genetic diversity of PCV3 in China.

## GENOME ANNOUNCEMENT

Porcine circoviruses are members of the genus *Circovirus* with two recognized species, porcine circovirus type 1 (PCV1) and PCV2. The full-length genome sequences of PCV1 and PCV2 are 1,759 and 1,767 to 1,768 bp long, respectively. PCV1 is not known to cause any obvious disease (1), but PCV2 was confirmed as a virulent pathogen that causes various syndromes in pigs (2). In October 2016, porcine circovirus type 3 (PCV3), with a genome sequence size of 2,000 bp, was considered a potential pathogen associated with multisystemic disease, porcine dermatitis and nephropathy syndrome (PDNS), and reproductive failure in pigs (3–6).

In September 2017, at a 250-sow pig farm in Guangxi Province, China, PCV3 strain CH/GX/1776D/2017 was isolated from the lungs of a weaned pig that suffered acute diarrhea and subsequently died. Tests for potential related pathogens, such as classical swine fever virus, porcine reproductive and respiratory syndrome virus, pseudorabies virus, PCV2, porcine epidemic diarrhea virus, transmissible gastroenteritis virus, porcine rotavirus, and porcine deltacoronavirus, were negative. However, the lung sample tested positive for PCV3 by the PCR method established in our laboratory. The PCV3 strain CH/GX/1776D/2017 whole-genome sequence was amplified by two segments using our PCR method. The genome of PCV3 strain CH/GX/1776D/2017 was assembled using the software program DNASTAR (version 7.0).

The full-length genome sequence of CH/GX/1776D/2017 was 2,000 nucleotides (nt) long, with a single-stranded circular genome. The PCV3 genome has two major open reading frames (ORFs), ORF1 and ORF2. The ORF1 gene (nt positions 216 to 1106) of PCV3 strain CH/GX/1776D/2017 encodes virus replication and initiation-related proteins. The ORF2 gene (nt positions 1980 to 1336 in the complementary negative strand) encodes a major structural protein. The functions of other predicted open reading frames require further study.

The complete genome of CH/GX/1776D/2017 has high nucleotide identity (98.0% to 99.6%) with other PCV3 strains that have been circulating in the United States, China, South Korea, Brazil, Italy, and Poland in recent years. The complete genome of CH/GX/1776D/2017 shares the highest nucleotide identity (99.6%) with PCV3/KU-1602 (GenBank accession number KY996338) in South Korea and the lowest nucleotide identity (98.0%) with PCV3/CN/GXLJ1/2017 (accession number MF405276). Phylogenetic analysis showed that PCV3 strain CH/GX/1776D/2017 has the closest phylogenetic relationships with the sequence of strain PCV3/KU-1604 (accession number KY996340) identified in South Korea in 2016. PCV3 strains CH/GX/1776D/2017 and PCV3-US/MO2015 (accession number KX778720) share similar genetic evolution and belong to the PCV3a subtype.

The genome sequence data of PCV3 strain CH/GX/1776D/2017 reported in this study will promote a better understanding of the epidemiology and evolutionary characterization of PCV3.

### Accession number(s).

The complete genome sequence of PCV3 strain CH/GX/1776D/2017 has been deposited in GenBank under the accession number MG550107.
